# TCM-Derived Natural Compounds Targeting the Gut Microbiota in Metabolic Dysfunction-Associated Steatotic Liver Disease: Gut–Liver Axis Mechanisms, Safety Considerations, and Translational Challenges

**DOI:** 10.3390/metabo16050342

**Published:** 2026-05-19

**Authors:** Huailin Deng, Ruiqiu Zhang

**Affiliations:** 1 Graduate School of Shandong University of Traditional Chinese Medicine, Jinan 250355, China; 2 National Institutes for Food and Drug Control, Chinese Academy of Medical-Sciences and Peking Union Medical College, Beijing 100730, China

**Keywords:** metabolic dysfunction-associated steatotic liver disease, gut microbiota, gut-liver axis, TCM-derived natural compounds, toxicological safety

## Abstract

The occurrence and development of metabolic dysfunction-associated steatotic liver disease (MASLD) are closely related to intestinal flora imbalance, intestinal barrier damage, and gut-liver axis dysfunction. Due to their multi-target regulatory effects and advantages in intestinal microecological intervention, Chinese herbal monomers have shown promising application prospects in the prevention and treatment of MASLD. However, basic research on their toxicity still lags behind, and issues related to safety and clinical translation urgently need attention. This article systematically reviews the research progress on how flavonoids, triterpenoids, alkaloids, and polysaccharides improve hepatic steatosis, inflammatory responses, and metabolic disorders from a toxicological perspective by reshaping the intestinal microbiota, repairing the intestinal mucosal barrier, regulating short-chain fatty acid and bile acid metabolism, and synergistically acting on signaling pathways such as TLR4/NF-kB, FXR, TGR5, SIRT1, and the NLRP3 inflammasome. Furthermore, by combining methods such as 16S rRNA sequencing, metagenomics, metabolomics, and multi-omics integration, the article analyzes their application value and limitations in toxicological mechanism research, and discusses the translational bottlenecks faced by Chinese herbal monomers in pharmacokinetics, bioavailability, quality standardization, targeted delivery, and toxicological safety. Existing evidence indicates that Chinese herbal monomers have a three-in-one intervention advantage of microecological remodeling-metabolic regulation-inflammation inhibition, but their long-term medication safety, toxic target organs, dose-effect/toxicity relationships, and potential drug interactions still need further clarification. This article aims to provide a systematic reference for the safety evaluation and clinical translational research of Chinese herbal monomers in the prevention and treatment of MASLD.

## 1. Introduction

Metabolic dysfunction-associated steatotic liver disease (MASLD) is a subtype of steatotic liver disease (SLD) defined by the presence of hepatic steatosis together with at least one cardiometabolic risk factor after exclusion of other dominant causes of steatosis. Its disease spectrum includes simple steatosis, metabolic dysfunction-associated steatohepatitis (MASH), liver fibrosis, cirrhosis, and even hepatocellular carcinoma [1]. MASLD is closely related to metabolic disorders such as obesity, type 2 diabetes, and dyslipidemia, and is one of the most prevalent chronic liver diseases worldwide [2]. However, there is still no universally accepted specific pharmacotherapy for MASLD, and lifestyle intervention remains the mainstay of management [3]. The existing drugs (such as pioglitazone, vitamin E, etc.) have limited efficacy and are accompanied by adverse reactions [4]. Therefore, there is an urgent need to develop novel safe and effective treatment strategies.

Intestinal microecology plays a key role in the occurrence and development of MASLD through the gut-liver axis [5]. Intestinal flora imbalance can lead to changes in the profile of metabolites such as short-chain fatty acids (SCFAs) and bile acids, as well as impaired intestinal barrier function, thereby promoting bacterial products such as lipopolysaccharide (LPS) to enter the portal vein, causing liver inflammation and lipid deposition [5]. Metabolites of gut microbiota (such as SCFAs, bile acids, tryptophan derivatives, etc.) can also affect liver metabolism and immune response by activating host nuclear receptors (such as FXR and PPAR) and G protein-coupled receptors (such as GPR43, etc.) [6,7]. Studies have shown that MASLD patients have reduced diversity of intestinal flora and imbalance of specific flora, such as increased ratios of Firmicutes and Bacteroidetes, which are considered to be related to the process of MASLD [8,9]. Therefore, improving the function of the “gut-liver axis” by regulating intestinal flora and its metabolites is regarded as an important strategy for the prevention and treatment of MASLD [5,6,7].

Traditional Chinese medicine (TCM) has the advantages of multi-target and overall regulation in the prevention and treatment of MASLD and has shown good application prospects [10,11]. In recent years, increasing evidence has suggested that TCM-derived monomers may improve MASLD by remodeling the gut microbiota, restoring intestinal barrier function, regulating microbial metabolites, and modulating host signaling pathways [12,13]. However, several important questions remain unresolved. First, current evidence is highly fragmented, with most studies focusing on single compounds or complex TCM formulations, while systematic comparison of monomers according to chemical class remains limited. Second, many studies report associations between microbiota changes and MASLD improvement, but causal evidence is still insufficient because functional validation using fecal microbiota transplantation, antibiotic intervention, germ-free models, or multi-omics integration remains scarce. Third, some mechanisms appear context-dependent or even contradictory [11,14]. For example, activation of intestinal FXR–FGF15/19 signaling has been reported to mediate the beneficial effects of some compounds, whereas inhibition of intestinal FXR may promote GLP-1 secretion and improve metabolic dysfunction in other settings. Fourth, the relationship among low oral bioavailability, intestinal microbial metabolism, pharmacological efficacy, and potential toxicity of TCM monomers remains poorly defined. Finally, long-term safety, dose–toxicity relationships, target-organ toxicity, quality standardization, and herb–drug interactions have not been sufficiently integrated into current mechanistic discussions [10,15]. Therefore, this review aims to synthesize available evidence on TCM-derived monomers targeting the gut microbiota in MASLD, classify representative compounds by chemical structure, compare their gut-liver axis-related mechanisms, and highlight unresolved mechanistic, methodological, toxicological, and translational issues. Based on the latest research results, this review systematically summarizes the role and mechanism of TCM-derived natural compounds in regulating intestinal flora to improve MASLD, focusing on the classification and discussion of representative studies of monomers with different structural types and elaborating their chemical characteristics, action targets, and research depth. At the same time, the challenges and prospects of pharmacokinetics, bioavailability, standardization, targeting, and toxicological safety of TCM-derived natural compounds from basic research to clinical translation were analyzed, and the advantages and limitations of intestinal microecological omics research technologies (16S sequencing, metagenomics, metabolomics, and multi-omics integration) were reviewed [14,16]. Finally, we will further focus on the regulatory network of key signaling pathways related to the “gut-liver axis” in the mechanism of TCM-derived natural compounds in order to provide theoretical reference for the study of traditional Chinese medicine intervention and new drug development of MASLD in the future.

In this review, the term “TCM-derived monomers” refers to chemically defined active constituents isolated from Chinese medicinal herbs, rather than whole TCM-derived natural compounds, decoctions, or compound prescriptions. These monomers include representative flavonoids, triterpenoids, alkaloids, and other purified active substances. Compared with complex herbal mixtures, TCM-derived monomers have clearer chemical structures, more controllable quality standards, and are more suitable for mechanistic, pharmacokinetic, and toxicological evaluations. Therefore, the present review focuses on monomeric or purified active constituents rather than complete TCM formulas.

The present review is not intended to be a purely toxicological review. Instead, it focuses on the gut microbiota-mediated pharmacological mechanisms of TCM-derived monomers in MASLD, with additional discussion of safety and translational challenges. Because gut microbiota affects MASLD mainly through the gut-liver axis, including intestinal barrier integrity, microbial metabolites, bile acid metabolism, endotoxin translocation, and inflammatory signaling, the gut-liver axis is used as the central mechanistic framework of this review.

Although recent narrative reviews have summarized the role of traditional Chinese medicine in modulating gut microbiota for MASLD, with emphasis on botanical drugs and polyherbal formulations, gut microbial diversity, intestinal barrier integrity, and bile acid/short-chain fatty acid metabolism. However, the present review provides a distinct and complementary perspective. Rather than broadly reviewing TCM prescriptions or complex herbal formulations, we focus specifically on TCM-derived monomers and organize the evidence according to chemical classes, including flavonoids, triterpenoids, alkaloids, and polysaccharide-related active substances. This classification enables a clearer comparison of structure-related mechanisms, representative compounds, and evidence depth. More importantly, our review emphasizes the toxicological and translational aspects of these monomers, including pharmacokinetics, bioavailability, quality standardization, targeted delivery, dose–toxicity relationships, target-organ toxicity, long-term safety, and potential herb–drug interactions. In addition, we discuss key gut-liver axis signaling pathways and microbiome-related omics technologies, aiming to provide a more mechanism-oriented and safety-focused reference for the development of TCM monomers targeting gut microbiota in MASLD.

## 2. Literature Search Strategy and Study Selection

This review was conducted as a structured narrative review rather than a formal systematic review or meta-analysis. Literature was searched in PubMed, Web of Science, Scopus, and CNKI using combinations of the following terms: “metabolic dysfunction-associated steatotic liver disease”, “MASLD”, “non-alcoholic fatty liver disease”, “NAFLD”, “gut microbiota”, “gut-liver axis”, “traditional Chinese medicine”, “Chinese herbal monomer”, “flavonoid”, “triterpenoid”, “alkaloid”, “polysaccharide”, “berberine”, “quercetin”, “naringenin”, “hesperidin”, “anthocyanin”, “ginsenoside”, “Ganoderma lucidum polysaccharide”, “mechanism”, and “toxicity”. The search mainly focused on studies published in English or Chinese.

Studies were included if they met the following criteria:

(i) the study investigated Chinese herbal monomers or monomer-like active substances;
(ii) the study was related to MASLD/NAFLD, hepatic steatosis, metabolic inflammation, or gut-liver axis dysfunction;
(iii) the study provided mechanistic evidence involving gut microbiota, intestinal barrier function, short-chain fatty acids, bile acid metabolism, inflammatory signaling, oxidative stress, or metabolic regulatory pathways; and
(iv) the study used in vitro, in vivo, or human clinical models.

Studies were excluded if they focused only on crude herbal prescriptions without identifiable active monomers, lacked mechanistic data, were unrelated to MASLD/NAFLD, or were review articles, editorials, letters, or conference abstracts without original data. Because the heterogeneity of compounds, models, dosages, and endpoints was high, a quantitative meta-analysis was not performed. Instead, the most relevant mechanistic and clinical studies were summarized narratively and presented in Table 1.

## 3. Research Progress of Traditional Chinese Medicine Monomer Regulating Intestinal Microecology to Improve MASLD

**Figure 1:**
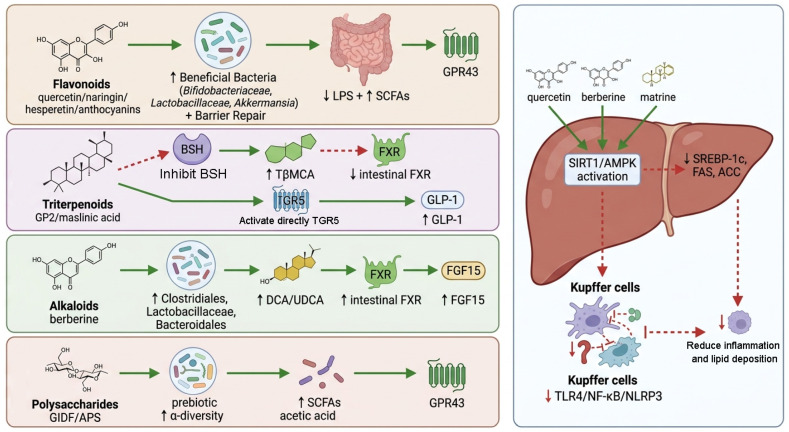
This diagram systematically displays the molecular pathways of flavonoids, triterpenoids, alkaloids, and polysaccharides, which regulate lipid metabolism and inflammatory response by reshaping the gut microbiota.

Recent evidence indicates that multiple categories of structurally heterogeneous monomeric compounds derived from traditional Chinese medicine can improve MASLD by reshaping the gut microbial environment. The major compound types investigated to date include flavonoids, triterpenoids, alkaloids, and polysaccharides. Therefore, this section summarizes representative monomers according to their chemical classification and discusses their structural attributes, principal targets, and available experimental evidence (Figure 1).

To clarify the relationship between the discussed monomers and traditional Chinese medicinal materials, we summarized the representative herbal sources of each compound or active substance in Table 2. In this review, TCM-derived monomers refer to chemically defined active constituents isolated from or enriched in Chinese medicinal herbs, rather than whole TCM-derived natural compounds or compound prescriptions. Therefore, the listed herbs indicate the traditional medicinal sources of these compounds, whereas the pharmacological mechanisms discussed in the following sections are mainly based on monomer-based or purified constituent studies.

### 3.1. Flavonoid Monomers

This section focuses on representative flavonoid-derived compounds with available MASLD-related evidence, including quercetin, naringin/naringenin, hesperidin/hesperetin, anthocyanins/proanthocyanidins, luteolin, and kaempferol [17,18].

Quercetin, a widely distributed polyhydroxyflavonol, has been shown to have significant lipid-lowering and liver-protective effects in MASLD animal models [19,20,21]. Quercetin can inhibit liver inflammatory signaling pathways by remodeling intestinal microecology and reducing endotoxemia [21]. Studies have found that quercetin can correct the imbalance of intestinal flora caused by a high-fat diet and reduce the release of endotoxin produced by harmful bacteria into the circulation, thereby blocking the activation of the TLR4/NF-κB signaling cascade mediated by endotoxin, thereby inhibiting downstream inflammasomes (such as NLRP3) and endoplasmic reticulum stress response. Finally, it blocks the dysregulated expression of genes related to liver lipid metabolism disorders and slows down the progression of MASLD [21]. At the same time, quercetin can also improve lipid metabolism by up-regulating the expression of key enzymes in metabolic pathways, such as activating the AMPK/SIRT1 pathway and down-regulating the expression of SREBP-1c and FAS genes related to lipid synthesis, thus inhibiting hepatic lipogenesis [19,20,22]. Studies in rats with HFD have confirmed that quercetin intervention for 12 weeks can significantly reduce liver triglyceride and total cholesterol accumulation, reduce hepatocyte ballooning and inflammation, and improve liver histology significantly. These effects are inseparable from the regulation of intestinal flora. Quercetin increases the abundance of beneficial intestinal bacteria (such as Bifidobacteriaceae and Lactobacillaceae), reduces the growth of endotoxin-producing pathogenic bacteria, and restores the intestinal barrier function, thereby reducing the inflow of inflammatory triggers such as LPS into the liver through the portal vein [21]. In conclusion, quercetin plays a significant role in liver protection through the intestinal microbiota-endotoxin-TLR4/NF-κB cascade pathway, which is one of the well-studied representatives of flavonoids [21].

Naringin and its metabolite, Naringenin, are another representative class of citrus flavonoids, which are widely found in traditional Chinese medicines such as citrus orange and tangerine peel and citrus fruits [23]. Studies have shown that naringin/hesperetin can reduce metabolic disorders induced by a high-fat diet and improve MASLD, this is one of the mechanisms is to protect the intestinal mucosal barrier and regulate the composition of intestinal flora [24,25]. Randomized controlled trials have shown that oral hesperetin 200 mg/day intervention for 4 weeks can reduce liver steatosis scores and improve body weight and blood lipid indicators in patients with MASLD [23,26]. Animal experiments have further revealed the mechanism. Hesperetin can enhance the expression of intestinal epithelial tight junction proteins, improve intestinal barrier function, and prevent the translocation of intestinal contents and microbiota products into the liver [25]. At the same time, it promotes the proliferation of symbiotic probiotics such as Lactobacillus and Bifidobacterium longum, inhibits the colonization of opportunistic pathogens, and corrects the imbalance of intestinal flora as a whole. The improvement of intestinal flora reduces the levels of inflammatory inducers such as LPS and inflammatory signals transmitted through the gut-liver axis, thereby inhibiting the excessive activation of the TLR4/NF-κB pathway in immune cells such as Kupffer cells in the liver and reducing the production of inflammatory factors [24,25,26]. In addition, hesperetin can also increase the level of intestinal SCFAs by promoting the growth of beneficial bacteria producing short-chain fatty acids (such as acetic acid and butyric acid), thereby activating the GPR43 receptor in colonic epithelial cells and indirectly increasing the secretion of glucagon-like peptide-1 (GLP-1) by colonic L-cells. It can improve insulin resistance and lipid metabolism [17,26]. These findings suggest that naringin and its metabolites have beneficial effects on MASLD from multiple links by maintaining intestinal microecological balance and intestinal barrier integrity, inhibiting gut-derived inflammatory signals, and are another typical flavonoid studied from the perspective of the “gut-liver axis”.

Anthocyanins are water-soluble pigment flavonoids found in blueberries, mulberries, etc. [27]. Anthocyanins are also abundant in some traditional Chinese medicine, such as purple jade plate. Anthocyanins have been shown to be hepatoprotective factors for MASLD intervention in recent years [27,28,29]. Clinical trials have shown that oral administration of anthocyanin extracts (such as grape seed extract, which is rich in anthocyanins) for 8–12 weeks can significantly reduce liver enzymes such as ALT and AST and the degree of hepatic steatosis in patients with MASLD [27,30]. In addition to its direct antioxidant and lipid-regulating effects, the underlying mechanism also involves the regulation of intestinal flora [28,29]. Proanthocyanidins (the aggregated form of anthocyanins) can selectively promote the proliferation of mucus-degrading bacteria, *Akkermansia muciniphila*, which is thought to help improve the intestinal mucosal barrier and promote mucus production in the intestinal epithelium, thereby enhancing the intestinal barrier function [28,29]. Proanthocyanidin treatment can restore the intestinal barrier damaged by a high-fat diet, reduce plasma endotoxin levels, and significantly reduce the LPS-TLR4-mediated inflammatory response in the liver [28]. In addition, anthocyanins have also been reported to up-regulate the bile acid receptor FXR and its downstream metabolic pathways, promote lipolysis and cholesterol clearance, and inhibit liver inflammatory signals, thus exerting hepatoprotective effects at multiple levels [31]. In summary, anthocyanins mediate bidirectional regulation of the intestinal environment and liver metabolism by affecting key intestinal flora (such as Akkermansia) and metabolites and have obvious benefits for MASLD.

Other flavonoid monomers, such as Kaempferol, Luteolin, and two hydrogen arbutus, etc., also show potential in MASLD [32,75,76]. For example, kaempferol can play a role by activating the autophagy pathway and inhibiting lipid deposition in hepatocytes [75,76]. It has dual regulatory functions in the AMPK/SIRT1 and AKT/mTOR pathways and is believed to promote the autophagic clearance of lipid droplets in hepatocytes, thereby reducing fatty liver [75,76]. Luteolin can improve insulin resistance and chronic low-grade inflammation by inhibiting TLR4-mediated inflammatory signals [32]. In general, flavonoids are the most active class of natural products in the intervention research of the MASLD gut-liver axis due to their structural diversity and multi-target characteristics [77]. Among them, the research basis of quercetin, naringin/hesperetin, and anthocyanins is relatively solid, which has gradually extended from animal experiments to preliminary clinical trials, showing good application prospects [21,23,27,29]. Flavonoids reduce the level of endotoxin and inflammation by enriching beneficial bacteria, inhibiting pathogenic bacteria, and enhancing the intestinal barrier. At the cellular signaling level, flavonoids activate metabolic pathways such as AMPK and PPARα and inhibit inflammatory pathways such as NF-κB. Thus, it has played a comprehensive role in regulating intestinal microecology and improving MASLD [18,21,25,26,32,76,77].

### 3.2. Triterpenoid Monomers

Triterpenoids are an important group of active ingredients in traditional Chinese medicine, including triterpenoid saponins, cardiac glycosides, and other subtypes. They are commonly found in ginseng, Glycyrrhiza, Ganoderma lucidum, gypenia pentyloides, and other traditional Chinese medicine [78,79,80]. In recent years, the role of triterpenes and their glycosides in regulating intestinal flora and improving metabolic function has attracted attention [33,34]. Some recent studies have revealed a new mechanism of triterpenes and their glycosides in alleviating MASLD through the gut-liver axis [33,34,35].

The gypenoside metabolite GP2 (2α-hydroxyprotopanaxadiol) is a representative case [35]. GP2 is a monomeric saponin produced by the metabolism of Gynostemma saponins in vivo, which has been proved to improve metabolic syndrome and fatty liver through the intestinal microbiota-bile acid-intestinal hormone axis [35]. Previous studies have shown that oral administration of GP2 to high-fat-fed mice reduces weight gain, improves glucose tolerance, and significantly reduces hepatic steatosis [35]. Mechanistically, GP2 inhibits the activity of bile saline lyase (BSH) of intestinal flora and adjusts the composition of intestinal flora, leading to the accumulation of conjugated bile acids in the intestine [36,37]. Among them, rat bile acid TβMCA (tauro-β-carnocholic acid, a primary bile acid binder) has an increased concentration in the intestine because it is not over-hydrolyzed, and TβMCA is a known FXR receptor antagonist in the small intestine [33]. The increase of TβMCA caused by GP2 antagonized intestinal FXR activity, thus relieving the inhibition of FXR on intestinal terminal L cells and inducing more GLP-1 production and secretion [38]. It was found that the plasma GLP-1 level of GP2-treated mice was significantly increased, and the expression of the Gcg gene (encoding proglucagon, the precursor of GLP-1) in colon tissue was also significantly up-regulated. Transplantation of fecal microbiota from GP2-treated mice to antibiotic-treated mice also replicated the above phenomenon (increased TβMCA, horizontal characteristics, and GLP-1 secretion), demonstrating that this effect was mediated by gut microbiota remodeling [38]. GP2 improved insulin sensitivity and hepatic lipid metabolism in mice by enhancing incretin hormones such as GLP-1 [35]. This study suggests that triterpene saponins can regulate intestinal endocrine signaling (FXR/GLP-1 axis) by regulating the metabolic function of intestinal flora (such as affecting the transformation of bile acids), and then exert beneficial effects on host energy metabolism and fatty liver [35,36,38].

Ginsenosides and their derivatives have also been extensively studied in metabolic diseases [39,40]. For example, the major saponins in ginseng, such as Rg1 and Rb1, are thought to have the potential to reduce fatty liver [39,40]. It has been reported that some structural analogues of ginsenoside (such as Cycloastragenol, the deglycosylation product of astragaloside) can activate the bile acid receptor TGR5, thereby stimulating GLP-1 secretion by intestinal L cells [40]. TGR5 is a G protein-coupled receptor distributed on enteroendocrine cells and liver macrophages. Its activation can promote the release of the incretin pancreatic hormone and inhibit the production of inflammatory factors by macrophages [41,42]. Maslinic acid is a pentyclic triterpene acid present in plants such as olive. Studies have found that it is one of the high-affinity ligands of TGR5 and can effectively activate TGR5 [43,44]. Maslinic acid can promote GLP-1 secretion after activating TGR5, and another triterpene, cycloastragalool, has also shown a similar effect [41,43,44]. In contrast, some isoflavones showed no significant effect on GLP-1 secretion, suggesting that the unique chemical structure of triterpenoids (containing polar groups such as hydroxyl, carboxyl, and carbonyl groups) may be the key to trigger TGR5 activity. In addition, triterpenoids often have anti-inflammatory properties, such as ursolic acid and oleanolic acid, which can reduce the production of macrophage pro-inflammatory factors and alleviate liver inflammatory injury [45,46,47]. Experimental evidence has shown that some triterpenes can reduce the abundance of intestinal endotoxin-producing bacteria, increase the proportion of short-chain fatty acid (especially butyric acid)-producing probiotics such as Lachnospiraceae and Akkermansia, and increase the fecal butyric acid concentration. Butyrate is an energy source for colonic epithelial cells and can also play an anti-inflammatory and improve lipid metabolism by acting on receptors such as GPR41/GPR43 [81,82]. With the increase of probiotics and butyric acid content, it can strengthen the intestinal epithelial barrier function and improve liver metabolism and inflammation through gut-liver axis signaling [81,82,83,84]. Therefore, triterpenoids are promising candidates with diverse mechanisms to regulate gut-liver signaling by directly activating receptors (such as TGR5, FXR, etc.) and indirectly regulate host metabolism by changing the composition of flora (increasing SCFA production and reducing endotoxin). Among these compounds, the study of GP2 has deepened our understanding of the novel mechanism of “inhibiting bile saline lyase—regulating bile acid group—enteroendocrine” [35,36,37,38]; however, maslinic acid and cycloastragalool highlight the unique role of triterpenes through the GPCR (such as TGR5)-intestinal hormone pathway [41,42,43,44,48].

### 3.3. Alkaloid Monomers

Alkaloids are another important family of monomers in traditional Chinese medicine (TCM) [49]. They often contain nitrogen atoms and are weakly basic, representing berberine, matrine, theophylline, etc. [50]. In the field of MASLD, especially Berberine (BBR) has been studied most dramatically [51]. Berberine is an isoquinoline alkaloid extracted from traditional Chinese medicine such as *Coptis coptis* and *Phelloberia amurensis* [50,51]. Due to its extremely low oral bioavailability, berberine is mainly retained in the intestinal lumen after ingestion, so its pharmacological effect is considered to be closely related to the intestinal tract [52]. Despite its low bioavailability, BBR has shown significant efficacy in improving metabolic syndrome, type 2 diabetes, and MASLD/MASH [53]. A study on MASH mice systematically revealed the mechanism of BBR’s action to improve liver disease through the gut microbiota-bile acid-intestinal FXR pathway [54].

In the study, high-fat and high-glucose-induced MASH mice were given BBR by gavage for 4 weeks, and liver steatosis and inflammatory cell infiltration were significantly reduced [55]. 16S rDNA sequencing analysis showed that BBR significantly adjusted the structure of the gut microbiome and increased the relative abundance of Clostridiales, Lactobacillaceae, and Bacteroidales. The imbalance of flora caused by a high-fat diet was corrected [56]. Meanwhile, bile acid analysis by ultra-high performance liquid chromatography-mass spectrometry found that the intestinal bile acid profile of mice was changed after BBR treatment: the relative levels of some secondary bile acids, such as deoxycholic acid (DCA) and ursodeoxycholic acid (UDCA) increased [55]. These bile acids are ligands or agonists of FXR receptors [57]. Among them, UDCA is a drug used in clinical practice for cholestatic diseases [58], while DCA can be converted from primary bile acids by intestinal flora [59]. According to this, researchers speculated that BBR changed the bile acid metabolic activity of intestinal flora and increased the production and reabsorption of FXR agonist bile acids [51]. Subsequent molecular detection confirmed that the expression of the FXR protein in the ileum tissue of the berberine treatment group was up-regulated, and the expression of the downstream target gene of FXR, FGF15, was also significantly increased [51]. FGF15 is a fibroblast growth factor secreted by the small intestine, which can flow back to the liver through the portal vein and act on the hepatocyte receptor FGFR4 to inhibit bile acid synthesis and regulate glucose and lipid metabolism [60]. In the BBR treatment group, due to the activation of FXR in the intestine, FGF15 secretion increased and played a role in inhibiting lipid synthesis and reducing inflammation after entering the liver [51]. Specifically, the expression of key enzymes for lipid synthesis (such as ACC and FAS) in the liver is decreased, the activity of the NF-κB inflammatory signaling pathway is decreased, and the level of proinflammatory cytokines is down-regulated [61]. On the contrary, in FXR knockout mice, the above improvement effects of BBR on hepatic steatosis and inflammation were greatly weakened or even disappeared, proving that the intestinal FXR-FGF15 axis is indispensable for BBR efficacy [52]. In addition, because BBR exists in high concentration in the intestinal lumen, it may also directly act on the intestinal flora itself, such as inhibiting the excessive proliferation of some Gram-negative bacteria, reducing the endotoxin produced by them, and reducing the inflammatory stimulation of the liver from the source [62]. In general, Berberine has achieved the effect of reducing fat, anti-inflammation, and alleviating MASH through a series of mechanisms of “regulating the structure of the flora, changing the bile acid metabolism profile, and activating the intestinal FXR-FGF15 signal” [52]. This study is an example of a Chinese medicine monomer acting through the gut-liver axis and explains why BBR can significantly improve metabolic diseases despite little oral absorption into the blood, as its main target is in the gut [50].

In addition to BBR, Oxymatrine, which is derived from the traditional Chinese medicine Kushen, is also a promising alkaloid monomer [63]. Matrine has some basic research on liver protection, anti-inflammation, and anti-fibrosis. For MASLD, studies have shown that matrine can reduce intrahepatic fat accumulation by activating the SIRT1/AMPK pathway and inhibiting the LXR/SREBP-1c lipid synthesis pathway in the liver [64]. Other animal experiments have shown that matrine can improve obesity and fatty liver in rats induced by a high-fructose and high-fat diet, and its effect is accompanied by the increase in the diversity of intestinal flora and the increase in the level of short-chain fatty acids. Although there are few reports on how matrine specifically affects the microbiota, it is speculated to be related to a certain degree of antibacterial activity and immunomodulatory effects. The role of other alkaloids such as anisodamine, berberine, and arecoline in MASLD needs more research, and BBR is the main representative at present. Berberine has moved from animal experiments to preclinical studies, and some small-scale clinical trials have shown that BBR has improved transaminase, blood lipids, and liver imaging indexes in MASLD patients [65]. However, its long-term efficacy and safety in humans still need to be verified by larger trials, which will be discussed in the clinical translation section of the subsequent chapter.

### 3.4. Polysaccharide Active Substances

Unlike small monomeric compounds, polysaccharides are macromolecular natural products, usually composed of multiple monosaccharides linked by glycosidic bonds [66]. Many traditional Chinese medicines, such as Astragalus membranaceus, Lycium barbarum, Ganoderma lucidum, and Poria cocos, are rich in polysaccharides. Dietary fiber and polysaccharides have a particularly significant effect on the gut microecology because they are often used as prebiotics in the gut by microbial fermentation to produce host-beneficial metabolites (e.g., SCFAs) [67]. Therefore, the regulation of intestinal flora by polysaccharides to improve MASLD is a highly important research direction.

Ganoderma lucidum polysaccharide and dietary fiber: Ganoderma lucidum is a medicinal and food fungus, and its extract has a long history of application in reducing blood lipids and protecting the liver [68]. In addition to triterpenoids (such as Ganoderma acid, etc.) in Ganoderma lucidum, polysaccharides and dietary fiber components are also considered to be the main functional substances [69]. A recent study used Ganoidum insoluble dietary fiber (GIDF) to intervene HFD-induced MASLD mice and achieved remarkable results. The results showed that compared with the non-intervention group, the mice that consumed GIDF had reduced liver weight and lipid accumulation, and liver histology confirmed that hepatocyte steatosis was significantly reduced and inflammatory cell infiltration was alleviated. Importantly, a control group with simultaneous administration of broad-spectrum antibiotics (the “AN-GIDF” group) was designed. The results showed that in the condition of gut microbiota depletion, the improvement of MASLD was partially impaired by GIDF [69]. These results suggest that the beneficial effects of GIDF largely depend on the existence of intestinal microecology [69]. 16S sequencing analysis showed that GIDF intervention significantly changed the composition of intestinal flora, and the α-diversity of intestinal flora in MASLD mice increased, and the β-diversity was closer to the normal group, indicating that GIDF reconstructs the disordered structure of intestinal flora and restores the intestinal ecological balance [70]. Specifically, the significant increase in the Firmicutes/Bacteroidetes (F/B) ratio induced by HFD was inhibited in the GIDF group. However, probiotic genera such as Lactobacillus, Blautia, Akkermansia, and Roseburia were significantly enriched in the GIDF group compared with the HFD group [71]. These bacteria are generally considered to be associated with healthy metabolic phenotypes in the literature. For example, Akkermansia can strengthen the mucosal barrier, and Blautia and Roseburia are short-chain fatty acid producing bacteria that can provide anti-inflammatory metabolites such as butyrate [72]. Correspondingly, the levels of SCFAs such as acetic acid, propionic acid, butyric acid, and valeric acid in the intestinal contents of the GIDF group were higher than those of the HFD group. The relative abundance of Akkermansia and Lactobacillus was negatively correlated with liver TG, ALT, edema, and other pathological indicators. However, it is positively correlated with some beneficial metabolites (such as L-cysteine, methylthioadenosine, etc.). This implies that the increase of these probiotics may be one of the key factors mediating the improvement of MASLD by GIDF [73]. On the other hand, GIDF also further promoted the positive regulation of the gut-liver axis by up-regulating intestinal mucosal defense and antioxidant/anti-inflammatory-related pathways [74]. It is worth mentioning that the same study also mentioned that the polysaccharide peptide in Ganoderma lucidum extract has been reported to reduce MASLD liver lipid accumulation by inhibiting fatty acid synthesis and activating the FXR-SHP/FGF15 pathway [85]. This suggests that the water-soluble polysaccharides and polysaccharide peptides of Ganoderma lucidum may also play a role through gut-liver signals such as FXR-FGF19 [85]. Overall, the Ganoderma lucidum polysaccharide/fiber study provides us with a good example of how natural dietary fibers can improve host metabolic disorders through microbiota metabolism. On the one hand, they provide fermentation substrates for probiotics and enhance beneficial metabolites such as SCFAs; on the other hand, microbiota adjustment reduced intestinal and liver inflammation, thereby significantly improving MASLD indicators.

Astragalus polysaccharide (APS): Astragalus membranaceus is a famous tonic traditional Chinese medicine, and its main active ingredient, Astragalus polysaccharide, has obvious immunomodulatory and metabolic protection effects [86]. In recent years, some studies have tried to apply astragalus polysaccharides to the prevention and treatment of MASLD and related liver diseases [86]. For example, Ge et al. developed colon-targeted astragalus polysaccharide nanoparticles (APs-CS/PT-NPs) for the study of preventing the transformation of MASLD to liver fibrosis-liver cancer. This nano-drug delivery system can protect polysaccharides from degradation in the upper digestive tract and target drug release to the colon, thus fully acting on intestinal flora. The results of animal experiments showed that oral administration of APs nanoparticles significantly reduced hepatic steatosis and inflammation in MASLD mice, and reduced the incidence of HCC. 16S sequencing showed that the intestinal microbial diversity of the mice treated with nanoparticles was restored; especially, the production of short-chain fatty acids (mainly acetic acid) was significantly enhanced. Transcriptome functional analysis further showed that acetic acid, as a key mediator, inhibited the NF-κB inflammatory pathway by activating GPR43 (an SCFA receptor on colon epithelial and immune cells), thereby playing an anti-inflammatory and anti-tumor effect [85]. This study proved the protective effect of increasing the level of gut-derived SCFAs on liver diseases from the side and also suggested that improving the efficacy of traditional Chinese medicine polysaccharides and achieving intestinal targeting through the modification of the delivery system were promising strategies. In addition to astragalus membranaceus, sea buckthorn polysaccharide, lycium berry polysaccharide, and other polysaccharides combined with APS also show synergistic liver protection in alcoholic and non-alcoholic fatty liver models, and the regulation of intestinal flora is involved behind it.

Other polysaccharides and prebiotics, such as dietary fiber, inulin, and fructooligosaccharides, are not directly derived from traditional Chinese medicine, but as functional food components, their mechanism is similar to that of traditional Chinese medicine polysaccharides. It is worth noting that traditional Chinese medicine compounds or decoctions often contain large amounts of polysaccharides, which play the role of “nourishing and benefiting bacteria” in the body, which may also be the reason why many compounds can improve metabolism as a whole. For example, changes in the ratio of intestinal Firmicutes to Bacteroidetes, the repair of mucosal barrier damage, and the adjustment of bile acid metabolism have been observed in the mechanism of the classic formula Yinchenhao decoction to improve MASLD. These alterations are most likely co-mediated by the crude polysaccharide components enriched in the prescription. In summary, polysaccharides have shown great potential in the intervention of MASLD by rebuilding intestinal commensal flora and providing beneficial metabolites, and their effects are more to “improve the internal environment” than to antagonize the enzyme or receptor directly, so they are often complementary with other TCM-derived natural compounds. In the future, a comprehensive interpretation of the effects of polysaccharide prebiotics on the microbiota and the host metabolic network through omics will help promote the application of such substances in metabolic liver diseases.

In summary, the active monomers from different structural types of TCM have all achieved representative results in regulating intestinal microecology to improve MASLD. Flavonoids focus on multi-target anti-inflammatory and anti-lipid (such as quercetin and naringin acting on the flora and inflammatory signals); triterpenoids have both receptor regulation and microbiota modification (for example, GP2 and maselin acid affect FXR, TGR5, and microbiota metabolism). Alkaloids, especially berberine, highlight the importance of the bile acid metabolic pathway. Polysaccharides comprehensively improve the composition of microbiota and the gut environment through prebiotic effects [74]. Due to the differences in chemical structure and mechanism of action, the research depth of various monomers is different: at present, there is relatively more clinical evidence for flavonoids and alkaloids (berberine, etc.), while the research on triterpenoids and polysaccharides is mostly in the stage of animal and mechanism exploration. However, taken as a whole, these studies collectively emphasize the central role of the gut-liver axis in the intervention of MASLD by TCM-derived natural compounds, that is, by improving the balance of intestinal flora, reducing harmful bacterial metabolites, and promoting the generation of beneficial signaling molecules, thus reducing liver lipids, anti-inflammation, and anti-oxidation at the systemic level. This understanding provides a scientific basis for the development of innovative therapies for MASLD based on intestinal microecological modulation.

## 4. The Regulatory Mechanism of Key Signaling Pathways in the Gut-Liver Axis

**Figure 2:**
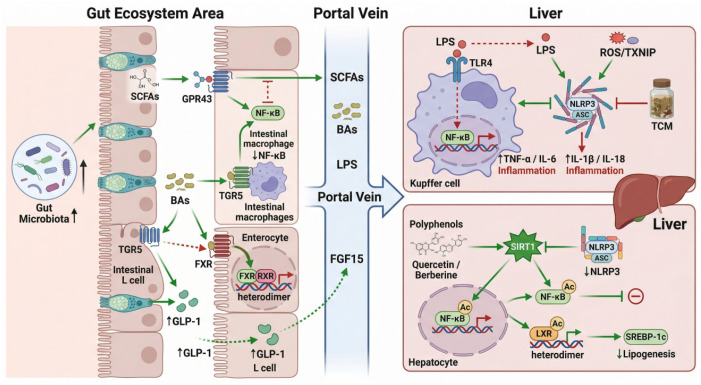
This diagram shows the five core molecular pathways regulating metabolism and inflammation in the “Gut Liver Axis”, reflecting the complex interaction between intestinal microbial metabolites, barrier function, and liver immune response.

The intestinal microecology communicates with the liver through multiple signaling pathways, and some of these “axes” play a crucial role in the occurrence and development of MASLD as well as the mechanism of action of TCM-derived natural compounds. The following text will delve deeper into how the main intestinal-liver axis-related pathways such as TLR4/NF-κB, FXR, TGR5, SIRT1, and NLRP3 inflammatory bodies are influenced by the intestinal microbiota and TCM-derived natural compounds and analyze the mechanism chains of these pathways in the process of improving MASLD (Figure 2).

### 4.1. TLR4/NF-κB Pathway

TLR4/NF-κB is a classic pro-inflammatory signaling pathway, playing a central role in the progression of MASLD from simple fatty liver to MASH [87]. TLR4 (Toll-like receptor 4) is mainly expressed on immune cells such as liver macrophages (Kupffer cells) and can recognize pathogen-associated molecular patterns such as LPS [88]. When the intestinal microbiota is imbalanced and the intestinal barrier is damaged, excessive LPS and other endotoxins enter the portal venous system and bind to TLR4 in the liver, activating the intracellular NF-κB transcription factor, inducing the expression of pro-inflammatory factors such as TNF-α and IL-6, leading to liver cell inflammation and damage [89]. Therefore, the over-activated TLR4/NF-κB pathway is regarded as one of the driving factors for the progression of MASLD to inflammatory hepatitis [90].

After regulating the intestinal microbiota with TCM-derived natural compounds, it is generally observed that the excessive response of the TLR4/NF-κB signaling pathway can be weakened [91]. This has been confirmed in various studies. The mechanism can be summarized into two levels:

One approach is to reduce the triggering signals by correcting the imbalance of the microbiota and repairing the intestinal mucosa, thereby reducing the entry of LPS and other ligand molecules into the liver [92]. As mentioned earlier, quercetin significantly reduces the activation of TLR4 in the liver of MASLD mice by increasing intestinal probiotics and reducing endotoxinemia. Similarly, naringin (bergapten) enhances intestinal barrier integrity, preventing the migration of intestinal bacterial products to the liver and reducing the stimulation of TLR4 at its source. Proanthocyanidins promote the growth of probiotics such as Akkermansia and repair the intestinal mucus layer, lowering plasma LPS levels, thereby blocking the inflammatory cascade mediated by LPS-TLR4. These all fall under the “treatment upstream” strategy, extinguishing the inflammatory trigger through microecological regulation.

The second is to antagonize signal transduction. Certain monomers of traditional Chinese medicine directly act on key nodes of the TLR4/NF-κB pathway, inhibiting the transmission of inflammatory signals [93]. For example, berberine has been found to reduce the phosphorylation of the NF-κB p65 subunit and downregulate the transcription of downstream pro-inflammatory genes while reducing bacterial LPS. The inhibition of NF-κB by BBR is related to its antioxidant effect. BBR reduces the level of ROS in the liver, thereby inhibiting the activation of the NLRP3 inflammasome and NF-κB by the TXNIP protein. In addition, many flavonoids such as resveratrol and luteolin have been reported to have the ability to block IκB enzyme activity and inhibit NF-κB nuclear translocation [94]. Therefore, even in the presence of certain inflammatory stimuli, TCM-derived natural compounds can indirectly inhibit NF-κB by enhancing antioxidant enzyme activity and activating upstream AMPK pathways. In addition, there is another type of G-protein-coupled receptor such as GPR43, which can also negatively regulate NF-κB through the cAMP/PKA pathway when activated by SCFAs produced by the microbiota. The aforementioned Huangqi polysaccharide nanoparticles increase the level of acetic acid, which is a typical mechanism. The activation of the acetic acid-GPR43 axis significantly inhibits the expression of inflammatory genes mediated by liver NF-κB. In summary, TCM-derived natural compounds achieve multi-target inhibition of the TLR4/NF-κB inflammatory pathway through dual regulation of microbiota and metabolites—reducing the production of “alarm signals” (LPS) and improving the system’s “tolerance” (inhibiting NF-κB activity), ultimately reducing the intensity of the inflammatory response. This is of great significance in preventing MASLD from deteriorating into MASH.

### 4.2. FXR Pathway

The Farnesoid X receptor (FXR) is a nuclear receptor that regulates bile acid, lipid, and glucose metabolism and is highly expressed in the liver and ileum [95]. The intestinal-hepatic FXR signaling axis is one of the important links through which the gut microbiota affects host metabolism, and it is also a popular target for drug intervention in MASLD [96,97]. The activation of FXR is generally believed to have the effects of reducing liver fat synthesis and improving insulin sensitivity, but excessive activation of intestinal FXR may inhibit the secretion of gut-promoting hormones, which is not conducive to metabolism. Therefore, in the treatment of MASLD, it is necessary to finely regulate the activity and tissue specificity of the FXR pathway [98,99].

In liver cells, FXR regulates genes related to bile acid and lipid metabolism. Activation of liver FXR can inhibit fatty acid synthesis mediated by SREBP-1c while enhancing FFA oxidation and VLDL excretion, which is beneficial for fatty liver [100]. Some single components of traditional Chinese medicine exert lipid-lowering effects by increasing liver FXR expression or activity. For instance, studies have shown that components such as eucommia lignan can enhance the expression of SHP downstream of liver FXR, reducing lipid accumulation in the liver. Although ursodeoxycholic acid (UDCA) is an exogenous bile acid, it is often combined with traditional Chinese medicine to activate FXR and improve liver steatosis [101].

In the ileum, FXR activation induces the secretion of FGF19/15 (human FGF19, mouse FGF15) [60]. FGF19 enters the liver and exerts systemic metabolic regulatory effects, including inhibiting hepatic insulin-mediated sugar production, etc. [102]. The berberine mentioned earlier is through increasing intestinal FXR agonistic bile acids (such as DCA and UDCA) to upregulate the FXR-FGF15 signal, thereby indirectly inhibiting liver lipid synthesis and inflammation [85]. This is a strategy of activating intestinal FXR. In this case, the moderate activation of intestinal FXR is beneficial for improving MASLD. This is consistent with the idea of treating MASLD with Obeticholic Acid (OCA, an artificial FXR agonist). However, there are also studies that adopt the opposite strategy, antagonizing intestinal FXR. The gypenoside GP2 increases TβMCA by inhibiting bile salt hydrolase, and TβMCA is an FXR antagonist, thereby weakening the intestinal FXR signal and promoting GLP-1 secretion. Since GLP-1 can improve insulin sensitivity and reduce fatty liver, this effect is beneficial for the overall metabolic syndrome. This seemingly contradictory phenomenon (sometimes activating FXR, sometimes inhibiting FXR) actually reflects the tissue dependence of the FXR pathway: activating liver FXR is beneficial for controlling lipid and bile acid homeostasis in the liver, but over-activating intestinal FXR can inhibit the secretion of GLP-1 by L cells, which is not conducive to glucose metabolism. Therefore, some scholars propose developing intestinal-selective FXR regulators or dual-target FXR regulatory strategies. For example, a possible scheme is to provide FXR activation when the liver needs it and avoid excessive stimulation in the intestine, or vice versa, depending on the specific pathology. The multi-target characteristics of single components of traditional Chinese medicine may have advantages here, as they often do not completely “open” a certain pathway like a single high-affinity drug, but rather moderately regulate multiple links. For example, berberine only partially increases intestinal FXR activity and simultaneously reduces inflammation, etc., making the overall situation more favorable, while GP2 moderately inhibits FXR and upregulates GLP-1, improving metabolism from another angle. Both ultimately improve MASLD. It can be predicted that different single components of traditional Chinese medicine may provide complementary mechanisms for the FXR pathway. Some are suitable for patients with low FXR activity and need enhancement, while others are suitable for cases with excessive FXR and need to be moderated.

### 4.3. TGR5 Pathway

TGR5, also known as GPBAR1, is another important member of the bile acid receptors. Unlike the nuclear receptor FXR, TGR5 is a membrane-bound G protein-coupled receptor. It is widely distributed in macrophages, intestinal endocrine cells, brown fat, etc., and plays a role in regulating inflammation and energy metabolism. Activation of TGR5 can increase intracellular cAMP, thereby inhibiting the NF-κB inflammatory response in macrophages, promoting GLP-1 secretion in intestinal L cells, and increasing energy consumption in muscles and fat. Therefore, TGR5 is considered a potential therapeutic target for MASLD and metabolic syndrome.

The connection between the gut microbiota and TGR5 mainly depends on the composition of bile acids. Certain secondary bile acids (such as lithocholic acid LCA, etc.) are strong agonists of TGR5, and the microbiota can influence TGR5 by producing these bile acids. Many single components of traditional Chinese medicine regulate the TGR5 pathway by altering the microbiota or acting as ligands directly. For example:

The aforementioned maslinic acid and cir-cinol are typical triterpenoid TGR5 activators. Their structures precisely match the characteristics recognized by TGR5 (multiple hydroxyl groups and multiple ring structures), and they can bind to the receptor to trigger downstream signals. On intestinal L cells, TGR5 activation will stimulate GLP-1 release; on liver macrophages, TGR5 can reduce the production of inflammatory cytokines [48]. Therefore, these triterpenoids can simultaneously address metabolic improvement (through GLP-1) and anti-inflammatory effects (by inhibiting Kupffer cell inflammation).

Ursodeoxycholic acid (UDCA) is also a TGR5 agonist (and also a weak FXR ligand). Although it is not a single component of traditional Chinese medicine, it is often used in combination with traditional Chinese medicine. UDCA can be increased through the action of the microbiome or through external supplementation. Activation of TGR5 helps promote bile secretion and alleviate inflammation in cases of cholestasis, and thus it is hypothesized to be beneficial in MASLD patients with bile-metabolism disorders.

Some polysaccharides or terpenoids of traditional Chinese medicine can indirectly increase the proportion of certain TGR5 ligand bile acids by enriching the bacteria that produce 7α-hydroxylated bile acid (such as Bacteroides species). This aspect of research is still relatively scarce and requires more attention.

The TGR5 pathway is worthy of attention in the action of TCM-derived natural compounds because, unlike FXR, TGR5 mainly mediates rapid membrane signals and does not involve gene transcription regulation. Its effect is relatively immediate [103]. For example, the bitter saponin (alkaloid), flavonoids, and triterpenoids contained in the GQD formula (a formula composed of kudzu root and cimicifuga root, etc.) jointly activate the bitter taste receptor and TGR5 and other GPCRs, immediately promoting the secretion of GLP-1, which is considered one of the reasons for GQD’s rapid reduction of mouse food intake and improvement of insulin sensitivity [48]. In the MASLD animal model, the activation of TGR5 by TCM-derived natural compounds often significantly reduces ALT, AST, and liver tissue inflammation score [104]. This indicates that its effect in protecting liver function is obvious. Of course, TGR5 activation may also have side effects, such as causing itching (activated TGR5 in skin neurons by bile acids) [105]. However, the activation efficacy of most TCM-derived natural compounds on TGR5 is moderate, and it is unlikely to cause strong adverse reactions.

Overall, the TGR5 pathway is an important link in the intestinal-liver axis that mediates anti-inflammatory and endocrine effects. By enhancing the gut microbiota that produce TGR5 agonists or directly providing agonist ligands, the active components of traditional Chinese medicine can effectively activate this pathway, thereby reducing liver inflammation, enhancing GLP-1 release, and regulating the dual abnormality of metabolic inflammation in MASLD. In the future, certain TGR5-activating components of traditional Chinese medicine are expected to be developed as therapeutic agents for metabolic syndrome and used in combination with FXR regulators to achieve more comprehensive efficacy.

### 4.4. SIRT1 Pathway

Silent Information Regulator 2-related enzyme 1 (SIRT1) is an NAD^+^-dependent histone deacetylase that plays a crucial role in regulating the metabolic homeostasis of the body and responding to stress [106]. SIRT1 regulates the activity of many transcription factors (such as PGC-1α, NF-κB p65, FOXO1, etc.) through deacetylation, thereby influencing lipid and glucose metabolism, inflammation, and mitochondrial function [107]. In the liver tissues of patients with MASLD, there is often a decrease in SIRT1 expression or activity, which is believed to be related to increased lipid synthesis in liver cells and enhanced inflammatory response [108].

Among the individual components of traditional Chinese medicine, many polyphenols have been proven to activate SIRT1. A classic example is resveratrol, which can mimic the effect of calorie restriction to upregulate SIRT1 and reduce liver fat deposition [109]. Specifically for the individual components covered in this review:

Flavonoids such as quercetin inhibit the lipid synthesis pathway by upregulating SIRT1. Studies have shown that quercetin can promote the phosphorylation of AMPK mediated by SIRT1, and the activation of AMPK further inhibits SREBP-1c to reduce lipid synthesis. At the same time, SIRT1 also deacetylated and inhibited NF-κB, resulting in decreased expression of pro-inflammatory genes. Therefore, quercetin intervenes in both metabolism and inflammation through SIRT1.

Berberine has been shown to activate the AMPK/SIRT1 pathway [53]. BBR increases intracellular NAD^+^ levels, thereby activating SIRT1, which deacetylates LXR and other transcription factors to inhibit lipid synthesis while reducing inflammatory responses by deacetylating NF-κB p65. Some reports point out that the effect of BBR on AMPK/SIRT1 is one of the important mechanisms of its anti-MASLD.

Matrine and hawthorn flavonoids have also shown the effect of enhancing SIRT1 expression in animal models. For example, after matrine increases SIRT1, it promotes PPARα-mediated β oxidation and inhibits the lipid synthesis signal of SREBP-1c, resulting in liver lipid reduction.

The link between the SIRT1 pathway and gut microbiota is mainly energy status: some metabolites of gut microbiota (such as resveratrol itself, which is derived from grape skin microbial fermentation) can affect host NAD^+^ level or SIRT1 expression [110]. In addition, when the diversity of microbiota is high, the host is in a low inflammatory state, which is also more conducive to the role of SIRT1. Therefore, TCM-derived natural compounds provide a good environment for the up-regulation of SIRT1 by improving the balance of flora and reducing oxidative stress. For example, Ganoderma lucidum dietary fiber reduces liver MDA level and increases GSH and other antioxidant indicators, which may reduce the inactivation of SIRT1 due to oxidative stress and essentially protect SIRT1 function. In addition, flora metabolites such as L-tryptophan can affect SIRT1 gene expression by activating the AhR pathway, which is indirectly regulated by TCM. In general, SIRT1 can be regarded as one of the key nodes connecting intestinal microflora, inflammation, and metabolism. TCM-derived natural compounds often achieve the effect of “killing two birds with one stone” by enhancing SIRT1 activity, which not only stimulates energy consumption, inhibits adipogenesis, but also reduces inflammatory response and oxidative damage. This makes SIRT1 a molecular target to explain the multiple effects of TCM and a therapeutic target that deserves further attention in MASLD.

### 4.5. NLRP3 Inflammasome Pathway

The NLRP3 inflammasome is an innate immune signaling complex that has been widely studied in recent years [111]. It is composed of NOD-like receptor protein 3 (NLRP3), aptamer protein ASC, and caspase-1 [112]. Once activated, NLRP3 inflammasomes promote the maturation of caspase-1, which catalyzes the release of inflammatory factors such as IL-1β and IL-18 and induces a programmed cell death called pyroptosis [113]. In MASLD, especially NASH, the NLRP3 inflammasome is considered to be an important promoter of hepatocyte inflammation and cell death [114]. NLRP3 can be activated by various stimuli such as high fatty acids, LPS and ROS [115]. Therefore, inhibition of this pathway is considered a potential strategy for anti-MASH.

Several studies have shown that TCM-derived natural compounds have an inhibitory effect on NLRP3 inflammasome, and this inhibition is closely related to intestinal microecology:

The role of berberine is most representative in this regard. Previous studies have shown that BBR treatment can significantly reduce the expression of the NLRP3 protein in the liver of MASH model mice, decrease the activity of caspase-1, and reduce the expression of pyroptosis executive protein GSDMD-N [116]. At the same time, BBR also reduced ROS levels and TXNIP (thioredoxin interacting protein) expression [53]. TXNIP is a molecule that connects ROS with NLRP3, and high ROS can dissociate TXNIP from thioredoxin and bind to activate NLRP3 [117]. The antioxidant effect of BBR weakens this activation pathway, thereby indirectly inhibiting the NLRP3 inflammasome through the ROS/TXNIP axis [118]. More interestingly, H_2_O_2_ treatment could partially reverse the inhibition of NLRP3 by BBR, further demonstrating the regulatory role of oxidative stress [56]. The inhibition of NLRP3 by BBR is also reflected in the reduction of the release of downstream inflammatory factors such as IL-1β and IL-18 and the protection of hepatocytes from pyroptosis [119]. Given that berberine can significantly alter gut microbiota composition and reduce blood LPS, these upstream effects undoubtedly also reduce the “first signal” required for NLRP3 activation (such as pro-IL-1β synthesis signal provided by TLR4-NF-κB) [120]. Therefore, BBR inhibits the NLRP3 inflammasome from the source (microbiota/LPS) to the signal (ROS/TXNIP), which is a model of immune regulation of the gut-liver axis.

Quercetin has also been reported to inhibit NLRP3 activity. It can reduce the LPS-mediated TLR4 stimulation and the expression of inflammatory body components, thereby reducing the production of IL-1β [121]. In high-fructose diet rats, quercetin reduced NLRP3 and caspase-1 levels in liver tissues, accompanied by improvement of gut microbiota, indicating a correlation between them.

Polyphenols such as resveratrol can also deacetylate and inhibit NLRP3 activity by activating SIRT1 [122]. Flavonoids enriched in hawthorn extract have been shown to down-regulate NLRP3 in alcoholic liver disease models.

The activation of the NLRP3 inflammasome is closely related to intestinal flora. A large number of studies have shown that intestinal LPS, bacterial membrane proteins, free fatty acids, etc., are one of the triggering factors of NLRP3. Chinese medicine monomers reduce the concentration of these danger signals by improving the intestinal ecology, thus curbing NLRP3 at the “first signal” stage. At the same time, the reduction of endogenous “second signal” (ROS) by anti-oxidation and up-regulation of mitochondrial function makes it difficult to fully activate NLRP3 [123]. Therefore, the multiple regulation of the NLRP3 inflammasome by TCM monomers in various key pathways makes them a powerful tool for the prevention and treatment of NASH inflammation and liver injury. This also explains why many traditional Chinese medicines are effective in reducing transaminase and inflammatory indicators, because they provide protection from the core link of cellular inflammation. In the future, some TCM-derived natural compounds may be considered in combination with existing anti-inflammatory drugs in order to achieve stronger intervention in the inflammation-fibrosis process related to the NLRP3 inflammasome.

In summary, TLR4/NF-κB, FXR, TGR5, SIRT1, NLRP3, and other pathways constitute an intertwined signal network on the gut-liver axis, which plays an important role in the pathogenesis of MASLD and the therapeutic effect of TCM-derived natural compounds. TCM-derived natural compounds are often not only targeted at a certain target, but also achieve multi-pathway synergistic effects through “network” regulation [124]. For example, berberine regulated both FXR-FGF15 (metabolism) and TLR4-NF-κB (inflammation) as well as NLRP3 (immunity); quercetin not only affected the intestinal barrier/TLR4 but also activated SIRT1/AMPK. Ganoderma lucidum polysaccharide not only inhibits NF-κB through SCFA-GPR43, but also involves FXR-FGF15 axis. Triterpenoids not only stimulate GLP-1 by TGR5 but also affect FXR by changing the composition of bile acids. This multi-target mode of action coincides with the requirement of “multi-hit” pathogenesis of MASLD. Therefore, understanding the regulation of these pathways from a systemic perspective is crucial for us to grasp the full picture of the action of TCM monomers.

## 5. Compound-Specific Microbiome and Multi-Omics Approaches for Studying TCM-Derived Natural Compounds in MASLD

Microbiome-related technologies are discussed here only in the context of MASLD research. In studies of TCM-derived monomers for MASLD, these methods are mainly used to determine whether changes in gut microbial composition and function are associated with MASLD-relevant outcomes, including hepatic lipid accumulation, insulin resistance, liver inflammation, intestinal barrier injury, bile acid metabolism, short-chain fatty acid production, endotoxemia, and fibrosis progression. Therefore, microbiome data should not be interpreted as isolated descriptive results, but should be integrated with MASLD-specific biochemical, histological, metabolic, and inflammatory endpoints. To avoid a purely methodological description, this section links each microbiome-related approach to representative TCM-derived natural compounds discussed in this review. For example, berberine is used to illustrate microbiota–bile acid–intestinal FXR signaling; quercetin is used to illustrate gut dysbiosis, endotoxemia, and TLR4/NF-κB-related inflammation; GP2 is used to illustrate microbiota-dependent bile acid transformation and FXR/GLP-1 regulation; and Ganoderma lucidum polysaccharide and Astragalus polysaccharide are used to illustrate intestinal fermentation, short-chain fatty acid production, and secondary gut–liver axis signaling. Therefore, the purpose of this chapter is not to summarize microbiome technologies in general, but to explain how these approaches can help clarify compound-specific mechanisms relevant to MASLD.

**Figure 3:**
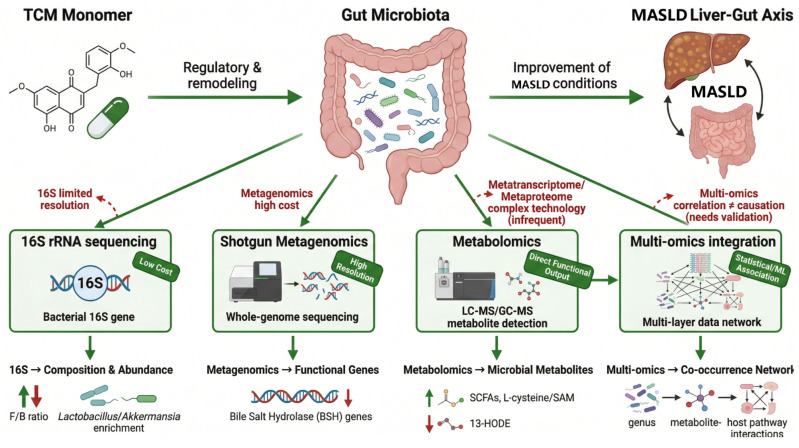
Application and methodological comparison of gut microbiome in revealing the mechanism of traditional Chinese medicine (TCM) monomer therapy for MASLD.

In order to further reveal the mechanism of intestinal microecology in the action of TCM-derived natural compounds, various omics techniques have been widely used to analyze the changes in the structure and function of intestinal flora. Each of these techniques has its own advantages and disadvantages, but their combination can provide a more comprehensive view. The following is a brief introduction to the commonly used intestinal microecoomics methods, their application value, and limitations in traditional Chinese medicine research (Figure 3).

### 5.1. 16S rRNA Gene Sequencing

This is the classic approach to studying the composition of gut microbiota, by amplifying and sequencing specific variable regions of the bacterial 16S rRNA gene to identify microbiota taxa in a sample [125]. Its advantages are relatively low cost, mature operation, suitability for large-scale sample comparison, and ability to provide information on the abundance of bacteria at phylum, family, genus, and other taxonomic levels [126]. In many MASLD-related studies, 16S sequencing was used to observe whether traditional Chinese medicine treatment corrected the phenomenon of reduced diversity of flora or increased proportion of harmful bacteria [127]. For example, the above-mentioned Ganoderma lucidum dietary fiber intervention experiment found that GIDF significantly reduced the F/B ratio of HFD mice and enriched beneficial bacteria such as Lactobacillus and Akkermansia. Quercetin, berberine, and other studies are also commonly used to analyze the changes of bacterial flora in 16S. However, the 16S method also has limitations, such as limited resolution, usually only to the level of genus or species identification, and the accuracy is limited by the completeness of the database [128]. Functional information cannot be provided directly, and only bioinformatics tools such as PICRUSt can be used to speculate on the function of the flora, but the accuracy is limited [129]. In addition, 16S sequencing is effective for bacteria, but not enough attention has been paid to other gut microorganisms such as fungi and viruses. In traditional Chinese medicine (TCM) studies, sometimes we are more concerned about functional changes rather than just taxonomic abundance, and 16S data should be interpreted in combination with other information.

In the context of the compounds reviewed here, 16S rRNA sequencing is most useful for determining whether a specific compound is associated with restoration of MASLD-related gut dysbiosis. For example, quercetin has been associated with correction of high-fat diet-induced microbial imbalance, enrichment of beneficial bacteria, reduction of endotoxemia, and attenuation of gut-liver inflammatory signaling. Berberine has also been reported to reshape gut microbial composition in metabolic liver disease models, which may contribute to changes in bile acid metabolism and intestinal FXR–FGF15/19 signaling. For Ganoderma lucidum polysaccharide, 16S sequencing can help determine whether enrichment of Lactobacillus, Akkermansia, Blautia, or Roseburia is associated with increased short-chain fatty acid production and improvement of hepatic steatosis. However, such 16S-based associations should not be interpreted as proof of causality unless supported by antibiotic treatment, fecal microbiota transplantation, germ-free models, or metabolite rescue experiments.

### 5.2. Shotgun Metagenomics

This method randomly sequenced the microbial genomes in the samples, obtained a large number of sequences, and assembled them for annotation. It can simultaneously identify bacteria, archaea, fungi, viruses and other microorganisms, and analyze their functional genes [130]. Shotgun metagenomics is particularly valuable for compounds whose proposed mechanisms depend on microbial enzymatic functions rather than only changes in bacterial abundance. For berberine, metagenomic analysis may help clarify whether changes in bile acid-transforming enzymes contribute to altered intestinal FXR–FGF15/19 signaling and subsequent improvement of hepatic lipid metabolism. For GP2, this approach may help determine whether inhibition of microbial bile salt hydrolase activity and accumulation of tauro-β-muricholic acid are linked to intestinal FXR modulation and GLP-1 secretion. For Astragalus polysaccharide and Ganoderma lucidum polysaccharide, metagenomics may identify functional enrichment of carbohydrate-active enzymes and short-chain fatty acid-producing pathways, thereby providing mechanistic support for their prebiotic-like effects in MASLD. The advantages of metagenomics are high resolution, species or even strain-level precision, and direct access to information on the functional potential of bacterial flora (such as metabolic enzyme genes, drug resistance genes, etc.) [131]. In the study of MASLD, metagenomics can help to discover bacterial species-level changes and functional associations with the disease [9]. For example, some studies found through metagenomic analysis that berberine decreased the abundance of choledocholic enzyme genes in the intestine, which implied that berberine reduced primary bile acid debinding and enhanced FXR activation. However, metagenomics is costly, data analysis is complex, and deeper sequencing is required to cover long-tailed species [132]. In addition, due to the high content of host DNA in human gut samples, preprocessing steps such as de-host sequences are required to increase the proportion of microbial sequences. For the study of monomers of traditional Chinese medicine, metagenomics can comprehensively present the changes of flora and the enrichment of metabolic pathways, which is very helpful to elucidate the mechanism. However, the sample size and funding of many traditional Chinese medicine-related trials are limited, and few of them have used metagenomics. In the future, with the further reduction of sequencing costs, metagenomics will play a greater role in revealing the interaction mechanism between traditional Chinese medicine and flora.

### 5.3. The Metatranscriptome/Metaproteome

In addition to the DNA level, researchers can also perform omics analysis on the RNA transcripts or proteins of intestinal microorganisms to understand the actual activity of the flora. For example, the metatranscriptome can reflect which genes are highly expressed under a specific treatment and thus infer the degree of functional metabolism. The metaproteome directly detects microbial protein products. They reflect real-time function more accurately than metagenomes. However, its technical requirements are higher, sample processing is complex, and quantification is not easy, so it is rarely used in the study of the mechanism of TCM-derived natural compounds.

### 5.4. Metabolomics

This is an important means to study the gut-liver axis from the perspective of metabolites. Metabolomics can be divided into the host serum/urine metabolome, liver tissue metabolome, and intestinal contents (feces) metabolome. For the study of microbiota, more attention has been paid to microbiota-derived metabolites (such as short-chain fatty acids, bile acids, and amino acid derivatives) in feces or intestinal contents [133]. Metabolomics uses platforms such as LC-MS, GC-MS, or NMR, which can detect the content changes of hundreds of small molecular metabolites [134]. In MASLD, this helps us to understand the effect of TCM monomers on the overall metabolic network. For example, through the metabolome, it can be found that a Chinese medicine increases SCFAs and reduces pro-inflammatory lipids (such as arachidonic acid metabolites), etc. These changes often correspond to the adjustment of flora [135]. Non-target metabolomics was applied in the above-mentioned study on dietary fiber of Linji, and it was found that GIDF up-regulated intestinal L-cysteine, S-adenosylmethionine (SAM), and other sulfur metabolism-related molecules, and down-regulated oxidized lipid metabolite 13-HODE. Combined with the correlation analysis of metabolomics and microbiota data, the mechanism can be further inferred. For example, the increased abundance of Akkermansia is positively correlated with the increase of some amino acid metabolites, suggesting that Akkermansia may promote beneficial metabolism [136]. The advantage of metabolomics is that it can directly reflect the functional results, but the disadvantage is that many metabolites are difficult to clearly attribute to the host or the source of the flora. Moreover, metabolic regulation is systematic, and it may not be possible to clearly understand the mechanism by looking at the changes of some metabolites alone. Thus, it is often necessary to associate metabolic alterations with specific microorganisms or pathways in combination with microbiota. For the compounds discussed in this review, metabolomics should be used to connect microbial changes with MASLD-relevant functional metabolites. In studies of berberine, bile acid profiling is particularly important for determining whether altered levels of deoxycholic acid, ursodeoxycholic acid, or other FXR-related bile acids are associated with improved hepatic steatosis and inflammation. In studies of GP2, targeted bile acid metabolomics can help verify whether changes in tauro-β-muricholic acid are linked to intestinal FXR inhibition and GLP-1 secretion. For Ganoderma lucidum polysaccharide, Astragalus polysaccharide, and Lycium barbarum polysaccharide, fecal short-chain fatty acids, especially acetate, propionate, and butyrate, should be measured together with intestinal barrier markers and hepatic lipid endpoints. For quercetin, naringin/naringenin, and hesperidin/hesperetin, metabolomics may also help distinguish whether the observed hepatic effects are associated with parent compounds, microbial metabolites, or downstream changes in bile acid and short-chain fatty acid metabolism.

### 5.5. Multi-Omics Integrated Analysis

Due to the difficulty of a single omics approach in fully describing the complex interactions between the gut and liver, the integration of multi-level data has become a trend [137]. Multi-omics analysis refers to the joint interpretation of 16S/microbiome data of the microbiota, metabolomics data and even host transcriptome/proteome data, and the identification of correlations among different layers of data through statistical and machine learning methods [138]. For instance, correlation networks or covariate models can be established to show the associations between specific bacterial genera and specific metabolites or liver gene expressions [139]. This is helpful for generating hypotheses, such as “the increase of a certain bacterium affects the X pathway in the liver by producing a certain metabolite”. In the study of the mechanisms of traditional Chinese medicine (TCM), multi-omics methods are particularly in line with the holistic view of syndrome differentiation and treatment: it can comprehensively analyze the multi-dimensional changes in the microbiota-metabolism-host caused by TCM and identify the possible main axes of action [140]. For example, network pharmacology combined with omics data can construct a “disease-gene-target-drug” network to reveal the multi-pathway regulation of TCM monomers [141]. As mentioned above, in the future, through the integration of systems biology, network pharmacology, and omics data, a more comprehensive understanding of the mechanism of TCM in treating MASLD can be achieved. The limitations of multi-omics analysis lie in the large volume of data, complex analysis, and the fact that correlation does not equal causation, which requires further experimental verification [142]. Therefore, multi-omics is usually used to propose mechanism hypotheses, and then targeted experiments are designed for verification. However, it is undeniable that the integration of multi-omics has greatly broadened our perspective on the interactions between TCM, microbiota, and the host, moving the research beyond a single path and truly towards the level of an overall system.

For MASLD research, multi-omics integration should be anchored to compound-specific and disease-specific endpoints. For example, an integrated study of berberine should connect gut microbial composition, bile acid profiles, intestinal FXR–FGF15/19 signaling, hepatic lipid synthesis genes, inflammatory markers, and liver histology. For quercetin, multi-omics analysis should link microbial restoration, endotoxin reduction, TLR4/NF-κB or NLRP3 inflammasome activity, oxidative stress, and hepatic triglyceride accumulation. For GP2, the key analytical chain should include microbial bile salt hydrolase activity, tauro-β-muricholic acid levels, intestinal FXR activity, GLP-1 secretion, insulin sensitivity, and hepatic steatosis. For Ganoderma lucidum polysaccharide and Astragalus polysaccharide, integration should focus on polysaccharide fermentation, short-chain fatty acid production, GPR43-related signaling, intestinal barrier integrity, and hepatic inflammation or fibrosis-related outcomes. Such compound-specific multi-omics frameworks would make the mechanistic conclusions more precise and reduce the risk of superficial associations.

In summary, the gut microbiome omics approach provides a powerful tool for elucidating the mechanism of action of individual components of traditional Chinese medicine. 16S sequencing rapidly sketches the profile of the microbiota, metagenomics deeply characterizes the functional potential of the microbiota, and metabolomics reflects the final functional output. The integration of multi-omics links multiple layers of information, providing a basis for establishing the mechanism network of the gut-liver axis. In specific applications, the appropriate method or combination of methods should be selected based on the research purpose and resources. For example, when focusing on whether a certain component can restore a healthy microbiota structure, 16S can be the first choice; if more concerned about functional metabolism, metabolomics or even metagenomics should be supplemented. It should be noted that any omics results should be interpreted with caution, as the intestinal ecosystem is extremely complex, and there is a need for repeated verification between data analysis and biological significance. For researchers of traditional Chinese medicine, while fully utilizing omics technologies, it is also necessary to combine traditional pharmacological methods to verify key links so as to convert big data discoveries into reliable mechanism explanations.

## 6. Compound-Specific Translational Challenges of TCM-Derived Natural Compounds Targeting the Gut-Liver Axis in MASLD

Although numerous TCM monomers have shown promising effects in experimental MASLD models, their clinical translation remains limited by several concrete and unresolved issues. Future studies should move beyond efficacy observation and address the following aspects in a more systematic manner: pharmacokinetic characterization, improvement of oral bioavailability, batch-to-batch quality control, gut- or liver-targeted delivery, standardized toxicological evaluation, and rigorous clinical validation. These issues are particularly important because many TCM monomers act locally in the intestinal lumen, undergo extensive microbial metabolism, or show low systemic exposure after oral administration. Therefore, clarifying the relationship among intestinal exposure, microbial transformation, systemic pharmacokinetics, efficacy, and toxicity will be essential for their development as MASLD interventions. These translational challenges should be discussed in a compound-specific manner because berberine, quercetin, naringin/naringenin, hesperidin/hesperetin, GP2, ginsenosides Rg1/Rb1, ursolic acid, oleanolic acid, maslinic acid, Ganoderma lucidum polysaccharide, Astragalus polysaccharide, and Lycium barbarum polysaccharide differ markedly in solubility, intestinal absorption, microbial metabolism, systemic exposure, liver distribution, and safety profile. Therefore, the translational value of each compound depends not only on whether it regulates a signaling pathway, but also on whether sufficient exposure can be achieved at the relevant intestinal or hepatic site of action.

### 6.1. Pharmacokinetics and Bioavailability

Many TCM-derived natural compounds have pharmacokinetic defects such as poor water solubility, low intestinal absorption rate, and obvious first-pass effect, which make their oral bioavailability low [143,144]. For example, berberine shows extremely low plasma exposure after oral administration, suggesting that its effects on MASLD may be primarily mediated by intestinal mechanisms, including modulation of gut microbiota, bile acid metabolism, and intestinal FXR–FGF15 signaling, rather than by high systemic exposure of the parent compound. Similarly, polysaccharide-related active substances are generally poorly absorbed and are more likely to function as prebiotic substrates that promote SCFA production and downstream gut-liver axis signaling. In contrast, some flavonoids and triterpenoids, such as quercetin, naringin/naringenin, hesperidin/hesperetin, kaempferol, and luteolin, may undergo intestinal absorption and microbial or hepatic metabolism, and their metabolites rather than parent compounds may contribute to the regulation of hepatic AMPK/SIRT1, NF-κB, FXR, TGR5, or NLRP3-related pathways. Therefore, future mechanistic studies should determine whether the parent compound or its metabolites reach the proposed target tissue at effective concentrations [145]. This “low bioavailability, high efficacy” phenomenon is partly attributed to the indirect effects mediated by gut microbiota, but it is also a challenge for achieving controlled drug delivery. On the one hand, the low absorption limits the systemic action of the drug and requires a higher dose to be effective. On the other hand, the large presence of active ingredients in the gut may trigger problems such as local irritation or tolerance of the flora. In order to improve the bioavailability and increase the exposure of drugs in the body, various new drug delivery systems have been proposed. For example, the use of nano-carriers, solid dispersions, liposomes and other technologies can significantly enhance the solubility and permeability of some flavonoids and alkaloids [146]. The aforementioned study of astragalus polysaccharide colon-localized nanoparticles is an innovative attempt to release the active ingredients at a specific site through ph-sensitive materials, which greatly improves the efficacy. In addition, structural modification and preparation of prodrugs are also commonly used strategies to improve absorption by changing physicochemical properties. In summary, improving the pharmacokinetics of TCM monomers not only helps to exert the systemic effects of TCM monomers, but also reduces the dose and potential toxicity, which is a key step in clinical translation.

### 6.2. Component Standardization and Quality Control

TCM-derived natural compounds are often extracted from natural products, and the purity and composition of raw materials may vary between different sources and batches [147]. Quality control requirements also differ among specific compounds. For berberine, purity, salt form, intestinal stability, and potential microbial conversion should be controlled because its main site of action is likely the gut lumen. For quercetin, naringin/naringenin, and hesperidin/hesperetin, quality assessment should distinguish glycosides, aglycones, and major metabolites, because microbial deglycosylation may influence bioavailability and activity. For ginsenosides Rg1/Rb1 and GP2, the relative abundance of parent saponins and transformed sapogenins should be carefully characterized, as microbial metabolism may determine their biological effects. For Ganoderma lucidum polysaccharide, Astragalus polysaccharide, and Lycium barbarum polysaccharide, molecular weight distribution, monosaccharide composition, glycosidic linkage pattern, purity, and batch-to-batch consistency are especially important because these properties may influence fermentation, SCFA production, and microbiota modulation. Clinical application requires clear ingredients and controllable quality, which poses a challenge to TCM-derived natural compounds with complex origins. Taking ginsenosides as an example, the content of each saponin in different varieties or cultivation methods of ginsenosides is different, so it is necessary to establish effective purification and detection methods to ensure the stable content of active ingredients in preparations [147]. In addition, most studies use lab-grade purified products, and how to achieve high-purity preparation and cost control on an industrial scale is also a problem. Fortunately, with the progress of chromatographic separation and crystallization technology, many TCM-derived natural compounds have been able to obtain more than 95% purity products (such as berberine, puerarin, etc., have been mass-produced in industry) [148]. However, for some components with low extraction amount or many structural analogues (such as some terpenoids and flavonoids), complete separation and purification are still difficult. To solve this problem, it is necessary to strengthen standardization and establish uniform extraction process and quality standards, including fingerprinting, content determination, and impurity limit, so as to ensure the consistency of each batch of preparation [149]. Standardized quality standards can also provide a basis for clinical dose conversion and efficacy evaluation. At present, a few TCM-derived natural compounds such as curcumin and quercetin have been developed in the form of health products or drugs, but most of them are still at the research level, and it is necessary to cooperate with the scientific research community to promote the standardization process.

### 6.3. Targeting and Delivery Strategies

The target organ of MASLD is the liver, but many TCM monomers exert their effects through the gut. So an interesting paradox is, how do you target both the gut and the liver? Delivery strategies should therefore be selected according to the likely site of action of each compound. For berberine, gut-retained or intestine-targeted formulations may be more rational than strategies designed only to increase systemic exposure because microbiota remodeling and bile acid-related intestinal signaling appear to be central to its MASLD-related effects. For quercetin, naringin/naringenin, hesperidin/hesperetin, kaempferol, and luteolin, formulation approaches that improve solubility, intestinal absorption, metabolic stability, or hepatic exposure may be needed if direct regulation of hepatic AMPK/SIRT1, NF-κB, or NLRP3 pathways is proposed. For GP2, ginsenosides Rg1/Rb1, ursolic acid, oleanolic acid, and maslinic acid, delivery studies should clarify whether sufficient intestinal or systemic exposure is achieved to modulate FXR, TGR5, GLP-1 secretion, or hepatic inflammatory pathways. For Ganoderma lucidum polysaccharide and Astragalus polysaccharide, colon-targeted delivery may be particularly relevant because their biological effects are closely associated with microbial fermentation and short-chain fatty acid production. Some compounds, such as berberine, act directly in the intestine without being absorbed, rather than entering the liver [52]; while others, such as quercetin, require systemic absorption to act on receptors in the liver nucleus. Future delivery strategies may require problem-specific analysis. For monomers that primarily target gut microbiota, colon-localized preparations, such as the aforementioned astragalus polysaccharide encapsulated in chitosan/pectin nanoparticles, can be considered to improve the influence on the microbiota through gut-site-directed release. Such a design not only improves the efficacy but also reduces the degradation of the drug by the upper digestive tract and improves the utilization efficiency. For components that need to enter the liver to function, liver-targeting delivery systems (such as cholesterol-binding nanoparticles, liver-targeting liposomes, etc.) can be used to enrich drugs for hepatocytes. Recently, some ligand-modified nanocarriers, such as GalNAc-modified siRNA, can efficiently uptake drugs into liver cells through receptor mediation [150]. Traditional Chinese medicine monomers can also learn from a similar concept to achieve precise delivery. Of course, any delivery system needs to consider immunogenicity and safety in humans, so balancing targeting and biocompatibility is also a challenge.

### 6.4. Preclinical Safety and Toxicological Assessments

Traditional experience suggests that many Chinese medicinal monomers are derived from natural plants, and their toxic and side effects are relatively small. However, in the field of modern medical research, it is still necessary to ensure its safety through rigorous dose-toxicity evaluation. Accumulation of toxicity or specific organ damage may occur with high doses or long-term administration of some compounds. For example, a large intake of berberine may cause gastrointestinal discomfort and constipation, and a certain dose of glycyrrhizic acid has the risk of increasing blood pressure and electrolyte disturbances [50,151]. This suggests that we cannot assume that natural is safe. Safety evaluation should also be compound-specific. For berberine, gastrointestinal tolerance, long-term effects on gut microbiota, and potential interactions with lipid-lowering, antidiabetic, or hepatoprotective drugs should be carefully assessed in MASLD patients, who often receive multiple medications. For quercetin, naringin/naringenin, hesperidin/hesperetin, kaempferol, and luteolin, safety assessment should consider dose, duration, metabolite exposure, and possible interactions with hepatic drug-metabolizing enzymes or transporters. For ginsenosides Rg1/Rb1, GP2, ursolic acid, oleanolic acid, and maslinic acid, liver, kidney, cardiovascular, and endocrine-related safety endpoints should be included when these compounds are proposed to regulate bile acid receptors, GLP-1 secretion, or lipid metabolism. For Ganoderma lucidum polysaccharide, Astragalus polysaccharide, and Lycium barbarum polysaccharide, attention should be paid to batch consistency, immune modulation, fermentation-related gastrointestinal effects, and long-term microbiota changes. Before clinical transformation, systematic acute, subchronic, and chronic toxicological tests should be carried out to investigate the effects on liver and kidney function, cardiovascular system, and nervous system. In addition, TCM monomers may interact with other drugs, such as berberine, which is known to inhibit some hepatic drug enzymes and thus affect the metabolism of statins. Such interactions should be taken seriously in MASLD patients with multi-drug sharing. In current studies, many studies only focus on drug efficacy and do not adequately report safety indicators. In the future, rigorous safety assessments should be introduced, including administration window, safe dose range, potential genotoxicity or carcinogenicity, etc. If available, safety trials conducted under standard GLP procedures would be preferable so that the data would be more convincing. For example, some scholars have pointed out that although a traditional Chinese medicine compound is believed to have few side effects, one should be more cautious in the design and observation of clinical trials due to its many components and complex mechanism of action. Similarly for monomers, there must be sufficient animal data to support the safety of their long-term use before large-scale human trials.

### 6.5. Clinical Evidence and Regulatory Registration

From basic research to clinical application, solid evidence of evidence-based medicine is ultimately needed. At present, the evidence for a considerable number of TCM monomers remains at the cellular and animal levels, and whether they are effective for humans is uncertain. Even for ingredients such as quercetin and berberine, which are supported by some small clinical trials, larger samples and more rigorously designed randomized controlled trials (RCTS) are needed to verify that the efficacy is reproducible, significant and safe. Future clinical trials should prioritize compounds with a clear link among pharmacokinetics, gut-liver axis mechanisms, and MASLD-relevant endpoints. For berberine, trial design should include not only liver enzymes and liver fat content but also gut microbiota composition, bile acid profiles, intestinal FXR-FGF15/19-related markers, and gastrointestinal safety. For quercetin, naringin/naringenin, and hesperidin/hesperetin, clinical studies should consider whether circulating metabolites and hepatic exposure are sufficient to support the proposed effects on lipid metabolism and inflammatory signaling. For GP2 and ginsenoside-related compounds, bile acid composition, GLP-1 secretion, insulin resistance, and hepatic steatosis should be evaluated together. For Ganoderma lucidum polysaccharide and Astragalus polysaccharide, trials should include fecal short-chain fatty acids, intestinal barrier markers, microbiota diversity, hepatic inflammation, and fibrosis-related outcomes. Such compound-specific trial designs would provide stronger evidence than general efficacy observations. In this process, it is also necessary to consider the appropriate patient population, dose, and dosing regimen and establish objective clinical endpoints (e.g., liver histological improvement, biochemical changes, etc.). However, the promotion of natural products into large-scale clinical trials often faces financial and regulatory difficulties. Compared with chemical drugs, natural monomers may not be attractive in terms of intellectual property and return on investment. In addition, regulatory approval is also a major threshold, and the management of herbal and supplement products in many countries or regions is different from that of prescription drugs. How to locate the identity of a TCM monomer (drug or dietary supplement) will also affect its clinical development path. In general, as a drug, it needs to undergo more rigorous clinical trials and review, but after approval, it can be used for specific indications. As a health product, the threshold is lower, but it cannot claim therapeutic efficacy. Developers need to decide the declaration strategy based on the importance and feasibility of the unit. In general, strengthening multi-center clinical research and accumulating high-quality evidence are the only ways to promote the transformation of these TCM-derived natural compounds. Some studies have mentioned that the number of registered clinical trials of traditional Chinese medicine in the treatment of MASLD is very limited, and most of them are animal studies. Although some compounds are effective in animals, they lack human evidence [50,151]. Therefore, more clinical investigators should be encouraged to participate in the future, and the promising monomers should be pushed to the stage of clinical trials to gradually answer questions about their efficacy and safety.

Despite many challenges, TCM-derived natural compounds still have unique prospects for clinical application. First of all, there is no specific drug for MASLD, while the characteristics of multiple targets and overall regulation of traditional Chinese medicine conform to the multi-factor pathogenesis of the disease, which can be used as a beneficial supplement to single-target Western medicine therapy [152]. Secondly, many monomers, such as quercetin and curcumin, are widely available and can be produced in large quantities at low cost, which provides the possibility for the development of popular products in the future. Moreover, TCM-derived natural compounds usually have fewer side effects and have safety advantages in the long-term management of chronic diseases [140]. With the development of science and technology, we can more accurately analyze the mechanism of action and action targets of TCM-derived natural compounds so as to optimize their structure or compatibility. For example, structural modification can improve activity and reduce toxicity, or the combination of multiple monomers can form “synergistic combinations” to make use of their strengths to make up for their weaknesses. In summary, interdisciplinary cooperation is needed to accelerate the translation of TCM monomers, including medicinal chemical modification, application of advanced pharmaceutical technology, and rigorous clinical validation, in the face of many limitations such as pharmacokinetics and safety. Only in this way can the potential molecules in the laboratory truly benefit MASLD patients.

Future research should therefore prioritize the following directions. First, pharmacokinetic studies should determine not only plasma exposure but also intestinal luminal concentration, microbial metabolites, hepatic distribution, and enterohepatic circulation of representative TCM monomers. Second, standardized quality-control systems should be established, including chemical fingerprinting, purity determination, impurity profiling, and stability testing. Third, advanced delivery systems, such as colon-targeted nanoparticles, pH-sensitive carriers, liposomes, and hepatocyte-targeted formulations, should be explored to improve local intestinal or hepatic exposure. Fourth, toxicological studies should include acute, subchronic, and chronic toxicity tests, as well as assessments of hepatotoxicity, nephrotoxicity, cardiotoxicity, reproductive toxicity, genotoxicity, and potential herb–drug interactions. Finally, future clinical trials should adopt randomized, placebo-controlled designs, enroll well-defined MASLD or MASH populations, and use objective endpoints such as liver fat content, liver enzymes, fibrosis markers, insulin resistance, gut microbiota profiles, bile acid metabolites, and long-term adverse events. These improvements will help transform mechanistic findings into clinically meaningful therapeutic strategies.

## 7. Conclusions and Prospects

Recent studies have fully demonstrated the crucial role of intestinal microecology in the onset and treatment of MASLD. The active components of traditional Chinese medicine, with their multi-target regulatory advantages, have shown great potential in improving MASLD by reshaping the balance of intestinal flora and the “intestinal-liver axis” signaling network. From flavonoids, alkaloids, triterpenoids, and polysaccharides, various components approach the process from different angles, either supplementing probiotics, strengthening the intestinal barrier, regulating bile acid metabolism, activating intestinal receptors, or enhancing the host’s antioxidant and anti-inflammatory capabilities, ultimately achieving the effects of weight loss, anti-inflammation, and liver protection [153,154]. Mechanism studies based on omics technologies have deepened our understanding of these processes and provided scientific basis for the development of these traditional Chinese medicine components.

However, for the actual application of individual Chinese herbal components in clinical practice, we still need to overcome many challenges. Future work should focus on the following aspects: firstly, conduct in-depth clinical trials to verify the efficacy and safety, select the most promising candidates, and determine the appropriate dosage schemes [140]; secondly, apply pharmacokinetics and pharmaceutics methods to improve the bioavailability and targeting of the components, enabling them to exert their maximum effect in the human body; thirdly, establish a complete quality control standard to ensure the controllability of the purity and batch-to-batch stability of the active ingredients derived from natural sources; fourthly, utilize network pharmacology and multi-omics data to discover new targets and pathways for the action of Chinese herbal components, achieving the overall integration of the action mechanism; Fifthly, explore the synergistic effects between components and between components and modern drugs, which may lead to the development of combination therapies to cover all pathological links of MASLD [155]. With the advancement of technology, we can reasonably expect that more efficient and safe Chinese herbal components, and their derivative drugs will enter clinical practice for the prevention and treatment of MASLD, bringing good news to patients with fatty liver who have no specific drugs.

In conclusion, this review emphasizes the significance of the “gut-liver axis” as a regulatory hub for MASLD and systematically elaborates on the multi-dimensional mechanisms by which TCM-derived natural compounds improve MASLD through regulating the intestinal microecology. In future research and applications, it is necessary to combine inheritance and innovation. On one hand, we should deeply explore the potential of classic Chinese medicines and their monomers; on the other hand, we should utilize modern omics and drug development technologies to enhance them. Only in this way can we continuously enrich our understanding of the pathogenesis of MASLD, expand the scientific connotation of traditional Chinese medicine theory, and ultimately transform traditional wisdom into effective weapons for the prevention and treatment of metabolic liver diseases, contributing a Chinese solution to the increasingly severe MASLD situation worldwide.

## Figures and Tables

**Table 1:** Representative studies on Chinese herbal monomers or monomer-like active substances targeting the gut-liver axis in MASLD/NAFLD.

Compound/Active Substance	Class	Model/Evidence Type	Main Outcomes	Proposed Gut-Liver Axis Mechanism	Refs.
Quercetin	Flavonoid	HFD-induced NAFLD/MASLD mice; hepatocyte steatosis models; in vivo/in vitro	Reduced hepatic lipid accumulation, inflammation, ballooning, and insulin resistance.	Remodeled gut microbiota; reduced endotoxemia/LPS; inhibited TLR4/NF-kB/NLRP3; activated AMPK/SIRT1.	[17,18,19,20]
Naringin/Naringenin	Flavonoid	HFD-induced NAFLD mice; overweight/obese NAFLD patients; in vivo/human	Improved lipid profile, hepatic steatosis, and metabolic parameters.	Restored gut microbial balance; protected intestinal barrier; reduced LPS-mediated inflammation; regulated SCFA signaling.	[21,22]
Hesperidin/Hesperetin	Flavonoid	HFD-induced NAFLD mice; NAFLD patients; in vivo/human	Reduced hepatic steatosis, liver enzymes, inflammation, and metabolic dysfunction.	Enhanced tight junctions; enriched Lactobacillus/Bifidobacterium; increased SCFA signaling; inhibited TLR4/NF-kB.	[23,24]
Anthocyanins/Proanthocyanidins	Flavonoid polyphenols	NAFLD patients; diet-induced obesity/NAFLD animal models; human/in vivo	Reduced ALT/AST, hepatic steatosis, and metabolic injury.	Increased Akkermansia; improved mucus barrier; reduced endotoxin translocation; modulated bile acid-FXR signaling.	[25,26,27,28,29]
Luteolin	Flavonoid	Diet-induced NAFLD rats; in vivo	Improved insulin resistance, inflammation, steatosis, and mucosal injury.	Restored intestinal barrier and microbiota balance; inhibited TLR4-mediated inflammatory signaling.	[30]
Kaempferol	Flavonoid	Type 2 diabetic fatty liver mice; hepatocyte lipid models; in vivo/in vitro	Reduced hepatic lipid deposition and metabolic disturbance.	Activated SIRT1/AMPK; promoted autophagic lipid droplet clearance; inhibited AKT/mTOR-related lipid accumulation.	[31,32]
Gypenoside metabolite GP2	Triterpenoid saponin metabolite	HFD-induced metabolic syndrome/fatty liver mice; in vivo	Reduced weight gain, improved glucose tolerance, and alleviated steatosis.	Remodeled microbiota; inhibited bile salt hydrolase; increased T-beta-MCA; regulated intestinal FXR and GLP-1.	[33,34,35,36]
Ginsenosides Rg1/Rb1	Triterpenoid saponins	NAFLD or obesity-related fatty liver animals; in vivo	Reduced lipid accumulation, lipid peroxidation, ER stress, and inflammation.	Activated AMPK-related metabolic regulation; reduced oxidative stress and inflammasome activation.	[37,38]
Maslinic acid/Ursolic acid/Oleanolic acid	Pentacyclic triterpenoids	Metabolic inflammation, diabetes, obesity, or fatty liver-related models; in vivo/mechanistic	Improved inflammation, lipid metabolism, glucose homeostasis, and liver injury indices.	Activated TGR5 or PPARalpha; promoted GLP-1; increased SCFA-producing bacteria in some models; inhibited NF-kB.	[39,40,41,42,43,44,45,46,47]
Berberine	Alkaloid	HFD-induced NAFLD/MASH rodents; NAFLD patients; in vivo/human	Reduced steatosis, inflammatory infiltration, serum lipids, transaminases, and metabolic dysfunction.	Remodeled microbiota; altered bile acids; activated intestinal FXR-FGF15/19; inhibited NF-kB and NLRP3.	[48,49,50,51,52,53,54,55,56,57,58,59,60,61,62,63,64]
Matrine/Oxymatrine	Alkaloid	Diet-induced fatty liver or metabolic disorder animals; in vivo	Reduced intrahepatic lipid accumulation and inflammatory injury.	Activated SIRT1/AMPK; inhibited LXR/SREBP-1c lipogenesis; potentially improved microbiota diversity and SCFAs.	[61,62]
Ganoderma lucidum polysaccharide/dietary fiber	Polysaccharide/dietary fiber	HFD-induced MASLD mice; in vivo, including antibiotic-treated controls	Reduced liver weight, lipid accumulation, steatosis, and inflammatory infiltration.	Increased microbial diversity; enriched Lactobacillus, Akkermansia, Blautia, Roseburia; increased SCFAs; improved barrier signaling.	[65,66,67,68,69,70,71,72,73]
Astragalus polysaccharide	Polysaccharide	MASLD-related liver injury, fibrosis, or hepatocarcinogenesis animals; in vivo	Reduced steatosis, inflammation, and disease progression.	Colon-targeted delivery remodeled microbiota; increased acetate; activated GPR43; inhibited NF-kB.	[73,74]
Lycium barbarum polysaccharide and other herbal polysaccharides	Polysaccharide	Fatty liver-related experimental models; mainly in vivo	Improved lipid metabolism, liver injury parameters, and microecological disturbance.	Prebiotic-like effects; increased SCFA-producing bacteria; repaired gut barrier; restored gut-liver axis homeostasis.	[64,65]

**Table 2:** Representative TCM-derived monomers and their corresponding Chinese medicinal herbal sources.

Compound/Active Substance	Chemical Class	Representative Chinese Medicinal Herbs or Herbal Sources	Notes
Quercetin	Flavonoid	*Sophora japonica* L./Sophorae Flos or Sophorae Fructus; *Crataegus pinnatifida*/Crataegi Fructus; *Ginkgo biloba*/Ginkgo Folium	Widely distributed flavonoid; not specific to one herb
Kaempferol	Flavonoid	*Ginkgo biloba*/Ginkgo Folium; *Sophora japonica*/Sophorae Flos; *Camellia sinensis*/tea-related medicinal material	Often coexists with other flavonoids
Luteolin	Flavonoid	*Lonicera japonica*/Lonicerae Japonicae Flos; *Chrysanthemum morifolium*/Chrysanthemi Flos; *Perilla frutescens*/Perillae Folium	Anti-inflammatory flavonoid found in several herbs
Naringin	Flavonoid glycoside	*Citrus aurantium*/Aurantii Fructus Immaturus; *Citrus reticulata*/Citri Reticulatae Pericarpium; pomelo-related citrus sources	Gut microbiota can hydrolyze naringin to naringenin
Naringenin	Flavonoid aglycone	Metabolite of naringin from citrus herbs such as Aurantii Fructus Immaturus and Citri Reticulatae Pericarpium	Often produced after intestinal microbial transformation
Hesperidin/Hesperetin	Flavonoid glycoside/aglycone	*Citrus reticulata*/Citri Reticulatae Pericarpium; *Citrus aurantium*/Aurantii Fructus or Aurantii Fructus Immaturus	Hesperetin is the aglycone form of hesperidin
Anthocyanins/Proanthocyanidins	Polyphenolic flavonoids	Grape seed-related preparations; berry-derived medicinal or edible materials; *Schisandra chinensis* and other polyphenol-rich herbs may contain related polyphenols	Some sources are medicinal-food homologous rather than classical single TCM herbs
Ginsenosides Rg1/Rb1	Triterpenoid saponins	*Panax ginseng*/Ginseng Radix et Rhizoma; *Panax notoginseng*/Notoginseng Radix et Rhizoma	Representative ginseng saponins
GP2/2α-hydroxyprotopanaxadiol	Triterpenoid sapogenin	Metabolite related to *Gynostemma pentaphyllum*/Gynostemmatis Herba saponins and ginseng-type saponins	Often discussed as a metabolite rather than a direct herbal constituent
Ursolic acid	Pentacyclic triterpenoid	*Crataegus pinnatifida*/Crataegi Fructus; *Ligustrum lucidum*/Ligustri Lucidi Fructus; *Eriobotrya japonica*/Eriobotryae Folium	Common triterpenoid in several medicinal plants
Oleanolic acid	Pentacyclic triterpenoid	*Ligustrum lucidum*/Ligustri Lucidi Fructus; *Hedyotis diffusa*/Hedyotidis Diffusae Herba; *Glycyrrhiza uralensis*/Glycyrrhizae Radix et Rhizoma	Frequently coexists with ursolic acid
Glycyrrhizic acid	Triterpenoid saponin	*Glycyrrhiza uralensis*/Glycyrrhizae Radix et Rhizoma	Safety concerns include pseudoaldosteronism and electrolyte disturbance at high exposure
Berberine	Isoquinoline alkaloid	*Coptis chinensis*/Coptidis Rhizoma; *Phellodendron chinense* or *Phellodendron amurense*/Phellodendri Chinensis Cortex or Phellodendri Amurensis Cortex; *Berberis* species	Low oral bioavailability; mainly gut-localized effects
Matrine/Oxymatrine	Alkaloids	*Sophora flavescens*/Sophorae Flavescentis Radix	Known for anti-inflammatory and hepatoprotective activities
Astragalus polysaccharide	Polysaccharide	*Astragalus membranaceus*/Astragali Radix	Mainly acts through intestinal fermentation and immune/metabolic modulation
Ganoderma lucidum polysaccharide/fiber	Polysaccharide/dietary fiber	*Ganoderma lucidum*/Ganoderma	Often acts through prebiotic effects and SCFA production
Lycium barbarum polysaccharide	Polysaccharide	*Lycium barbarum*/Lycii Fructus	Medicinal-food homologous polysaccharide source
Poria cocos polysaccharide	Polysaccharide	*Poria cocos*/Poria	Representative fungal polysaccharide in TCM

## Data Availability

No new data were created or analyzed in this study.
